# The Dietary Inflammatory Index Is Associated with Subclinical Mastitis in Lactating European Women

**DOI:** 10.3390/nu14224719

**Published:** 2022-11-08

**Authors:** Myriam C. Afeiche, Alison Iroz, Frank Thielecke, Antonio C. De Castro, Gregory Lefebvre, Colleen F. Draper, Cecilia Martínez-Costa, Kirsti Haaland, Giovanna Marchini, Massimo Agosti, Magnus Domellöf, Thameur Rakza, Maria José Costeira, Mireille Vanpee, Claude Billeaud, Jean-Charles Picaud, Daryl Lim Kah Hian, Guimei Liu, Nitin Shivappa, James R. Hébert, Tinu M. Samuel

**Affiliations:** 1 Nestlé Institute of Health Sciences, Nestlé Research, Société des Produits Nestlé S.A., Vers-chez-les-Blanc, 1000 Lausanne, Switzerland; 2 Nestlé Institute of Health Sciences, Nestlé Research, Société des Produits Nestlé S.A., 1015 Lausanne, Switzerland; 3 Department of Health Promotion, Swiss Distance University of Applied Sciences, 8105 Regendorf, Switzerland; 4 T2 Bene Ltd., 4123 Allschwil, Switzerland; 5 SAS Institute Pte Ltd., Singapore 078881, Singapore; 6 Crown Bioscience, San Diego, CA 92127, USA; 7 PhenomX Health LaForge, Fondation EPFL Innovation Park, 1015 Lausanne, Switzerland; 8 Hospital Clínico Universitario, University of Valencia, 46010 Valencia, Spain; 9 Department of Global Health, Oslo University Hospital, 0450 Oslo, Norway; 10 Department of Neonatology, Karolinska University Hospital, 171 76 Stockholm, Sweden; 11 Neonatal Intensive Care Unit, Woman and Child Department, Del Ponte Hospital, Insubria University, 21100 Varese, Italy; 12 Department of Clinical Sciences, Umeå University, 901 85 Umeå, Sweden; 13 Department of Obstetrics, Lille University Hospital, 59037 Lille, France; 14 Life and Health Sciences Research Institute (ICVS), School of Medicine, University of Minho, 4710-057 Braga, Portugal; 15 Life and Health Sciences Research Institute (ICVS) and Biomaterials, Biodegradables and Biomimetics (3B’s), PT Government Associate Laboratory, 4710-057 Braga/Guimarães, Portugal; 16 Department of Neonatology, Senhora da Oliveira Hospital, 4835-044 Guimarães, Portugal; 17 Department of Pediatrics, Astrid Lindgren Children’s Hospital, Karolinska University Hospital, 171 64 Stockholm, Sweden; 18 Department of Women’s and Children’s Health, Karolinska Institutet, 171 77 Stockholm, Sweden; 19 Hôpital des Enfants, CHU Pellegrin, 33076 Bordeaux, France; 20 Division of Neonatology, Hôpital de la Croix-Rousse, 69004 Lyon, France; 21 CarMen Laboratory, INSERM U1060, INRA 69221, INSA Lyon, Claude Bernard University Lyon 1, 69000 Lyon, France; 22 Institute for Infocomm Research, Agency for Science, Technology and Research, Singapore 138632, Singapore; 23 Department of Epidemiology and Biostatistics and the Cancer Prevention and Control Program, Arnold School of Public Health, University of South Carolina, Columbia, SC 29208, USA; 24 Department of Nutrition, Connecting Health Innovations LLC, Columbia, SC 29201, USA; 25 Nestlé Product Technology Center-Nutrition, Société des Produits Nestlé S.A., 1800 Vevey, Switzerland

**Keywords:** lactating women, dietary inflammatory index, subclinical mastitis, multicenter longitudinal cohort

## Abstract

Subclinical mastitis (SCM) is an inflammatory state of the lactating mammary gland, which is asymptomatic and may have negative consequences for child growth. The objectives of this study were to: (1) test the association between the dietary inflammatory index (DII^®^) and SCM and (2) assess the differences in nutrient intakes between women without SCM and those with SCM. One hundred and seventy-seven women with available data on human milk (HM) sodium potassium ratio (Na:K) and dietary intake data were included for analysis. Multivariable logistic regression was used to examine the association between nutrient intake and the DII score in relation to SCM. Women without SCM had a lower median DII score (0.60) than women with moderate (1.12) or severe (1.74) SCM (*p* < 0.01). A one-unit increase in DII was associated with about 41% increased odds of having SCM, adjusting for country and mode of delivery, *p* = 0.001. Women with SCM had lower mean intakes of several anti-inflammatory nutrients. We show for the first time exploratory evidence that SCM may be associated with a pro-inflammatory diet and women with SCM have lower intakes of several antioxidant and anti-inflammatory nutrients.

## 1. Introduction

Subclinical mastitis (SCM) is an inflammatory state of the lactating mammary gland, which is asymptomatic and has the potential to change the composition of human milk (HM), progress to mastitis, and negatively affect child growth [1,2,3,4]. In mastitis, free radicals are released and the total antioxidant capacity in milk is decreased [5]. Vitamin E and selenium are especially important dietary antioxidants because of their ability to neutralize oxidants, thereby suppressing the progression toward increased production and deployment of pro-inflammatory cytokines [6,7]. From bovine studies, it is known that deficiencies in certain vitamins and trace minerals, before and after calving, are associated with an increased incidence of mastitis [8]. Furthermore, administration of vitamin E, selenium, zinc, vitamin A, vitamin C, β-carotene, and copper supplements has been shown to hasten recovery from mastitis [9].

In general, dietary nutrients play a central role in regulating inflammation. A high consumption of refined grains, simple carbohydrates, and red meat has been correlated with higher concentrations of C-Reactive Protein (CRP) and interleukin-6 (IL-6), important biomarkers of inflammation [10]. In contrast, lower levels of inflammation have been observed among people adhering to either a typical Mediterranean diet (low in red meat and butter, and high in whole grains, fish, fruit, and green vegetables, with moderate alcohol and olive oil intake) [11] or to other diets high in fruit and vegetables [12]. Vitamin D deficiency, or a low status of, have been associated with increased inflammation [13]. Omega 3 polyunsaturated fatty acids (n–3 PUFAs), vitamins A, D, C, and E, folic acid, and selenium have been shown to exert anti-inflammatory effects [14]. Diets rich in n–3 fatty acids such as eicosapentaenoic acid (EPA) (20:5n-3), docosahexaenoic acid (DHA) (22:6n-3), and α-linolenic acid (18:3n-3) have been shown to confer protective effects by modulating inflammatory functions in various immune cells [15]. Other nutrients such as β-carotene [16], vitamin E [17], magnesium [18], and vitamin C [19] are linked to lower levels of inflammation.

The dietary inflammatory index (DII^®^) is a tool to assess diet-associated inflammation and was published in 2009 [20]. The literature-derived DII, which is in its second version [21], is widely used in epidemiological research. Construct validity for the DII has been demonstrated in over 40 studies in various populations, where it has shown significant associations with elevated high-sensitivity CRP, IL-6, and tumor necrosis factor in a number of non-communicable diseases [22,23,24,25]. Currently, no data exist on the relation between DII, maternal dietary intake, and SCM. The objectives of this study were to: (1) test whether an association exists between the inflammatory potential of the diet, using the DII, and SCM during early lactation; and (2) determine if there are differences in the intakes of antioxidant and anti-inflammatory nutrients between women with and without SCM. 

## 2. Materials and Methods

### 2.1. Study Population

‘ATLAS’ is a longitudinal, observational study that was conducted across 6 European countries, including Sweden, Spain, Portugal, Norway, Italy, and France, between December 2012 and January 2016 [4]. The investigational sites for this study were public hospitals where a flyer was posted in the maternity waiting room. Pregnant women were recruited mostly during the last trimester of pregnancy. Participant inclusion criteria were: being 18 to 40 years of age when enrolling in the study; having a pre-pregnancy body mass index (BMI) between 19 and 29 kg/m^2^ inclusive; decision to exclusively breastfeed child for up to 4 months—meaning no other foods or liquids were provided to the infant, including water; and willingness to provide signed informed consent. Exclusion criteria were: taking part in another clinical study 4 weeks prior to starting this study; having twins; diseases or medical conditions such as abnormal conditions of pregnancy (e.g., hypertension), diabetes, heart problems; medical condition that precludes breastfeeding or collection of HM samples or for which breastfeeding is not indicated; celiac disease, anorexia, or bulimia; taking medications for any metabolic or cardiovascular disease treatment; and non-compliance with the study procedures.

Trained staff (including certified research nurses and assistants) collected maternal demographics, anthropometry, and medical history. All data were immediately entered into a secured web-based database (Medidata Rave edc 5.6.4., Medidata Solutions, Inc., a Dassault Systèmes company, New York, NY, USA). Three hundred and five women were screened. Data on dietary intake and SCM were available for 185 lactating women. Those with insufficiently robust dietary information (identified in a two-step process) were removed from the analysis. First, outliers of energy intake were identified as less than 1074 kcal or more than 4776 kcal as suggested elsewhere [26]. Second, participants with only 1 completed food diary day were removed from the analysis. The final sample size comprised of 177 women.

All methods were implemented in agreement with the relevant local and institutional guidelines and regulations. Women signed a written informed consent form to participate in the study after obtaining explanations and reading and understanding the purpose and the objectives of the study in their respective local languages. The study was approved by the institutional and local ethical boards for each center (Sweden: Regionala etikprövningsnämnden Umeå. Approval number No 2012-295-31M; Date of access 12 October 2012; Spain: Comité Ético de Investigación Clínica Del Hospital Clínico Universitario de Valencia. Approval number No 84; Date of access 13 July 2012; Portugal: Comissao de Etica do Centro Hospitalar Sao Joao, Comissao de Etica para a Saude do Hospital de Braga, Conselho de Administracao do Centro Hospitalar de Alto Ave. Approval number N-A; Date of access PRT001 Porto: Amdt 1 20 September 2013, PRT002: 3 December 2012, and PRT003: 14 September 2012; Norway: Regionale Komiteer for Medisinsk og Helsefaglig Forskningetikk. Approval number No 2012/916; Date of access 20 December 2012; Italy: Comitato Etico Ospedale di Circolo Fondazione Macchi. Approval number No 1211; Date of access 12 June 2012; and France: Comité de Protection des Personnes Sud-Ouest et Outre Mer III. Approval number No 2012-A00142-41; Date of access 30 May 2012) and was registered at ClincalTrials.gov with identifier NCT01894893.

### 2.2. Collection of Human Milk Samples

Following a standardized protocol for all participants, milk samples were obtained using an electric breast pump (Medela Symphony, Baar, Switzerland) at 11:00 h ± 2:00 h to avoid circadian influence. HM was collected from the same breast for the entire study for each mother. They were asked to empty the breast manually in the previous feed. Aliquots of 5–10 mL of colostrum at visit 1 (V1 0–3 days postpartum) were taken and 10–40 mL HM for all other time points were reserved for further analyses. Samples were first frozen at −18 °C until delivery to the Nestlé Research Center (Lausanne, Switzerland) and then stored at −80 °C for further analysis.

### 2.3. SCM Assessment

Milk samples were analyzed for sodium (Na) and potassium (K) using inductively coupled plasma mass spectrometry (ICP-MS), PerkinElmer model NexION 300 (Perkin Elmer, Waltham, MA, USA) as previously published [4]. SCM was assessed in early lactation, at visits 1 (V1) between 0 and 3 days postpartum, visit 2 (V2) 17 days postpartum ±3 days, and at visit 3 (V3) 30 days postpartum ±3 days. A composite variable was created if SCM was detected, and defined as having a sodium potassium ratio (Na:K) in HM higher than 0.6, at any of the three visits. Moderate SCM was defined as Na:K ratio between >0.6 and ≤1.0, while severe SCM as Na:K ratio >1.0 [27]. 

### 2.4. Dietary Intake Assessment

Food diaries were collected at V2 and V3. At each visit, participants provided a 3-day food diary. Food consumed, in grams, were averaged across the 3 days and 2 visits. In a second step, the average of foods in grams consumed was used to calculate the DII per participant (described in the next section). Dietary information was then transformed into daily nutrient intakes using the Nutrilog software (Nutrilog, Marans, France) and the 2017 version of the French food composition table (CIQUAL). 

### 2.5. DII Assessment Method

The procedure used to calculate the DII scores has been described previously [21]. In summary, using a thorough literature review of peer-reviewed articles published from 1950 to 2010, 1943 articles that assessed the role of 45 food macronutrients, micronutrients, flavonoids, and individual food items on tumor necrosis factor-alpha (TNF-α), interleukins, and high-sensitivity CRP were identified. The DII score was calculated in the laboratory of Dr. James Hébert, as previously described [21]. Of the 45 food parameters used to create the DII score, we were able to estimate 27 as the following ones could not be measured with the food diaries and food composition table employed in this study: trans fat, caffeine, eugenol, ginger, garlic, onion, saffron, turmeric, green/black tea, flavones, flavan-3-ol, flavonols, anthocyanidins, flavonones, isoflavones, thyme/oregano, pepper, and rosemary.

The z-score for each of the 27 food parameters was calculated based on the world average and standard deviation. A proportion (i.e., values from 0 to 1) for each food parameter was calculated then doubled and ‘1’ was subtracted to achieve a symmetrical distribution with values centered on 0 (null) and bounded between −1 (maximally anti-inflammatory) and +1 (maximally pro-inflammatory). The centered proportion for each food parameter was then multiplied by its respective ‘overall food parameter-specific inflammatory effect score’ to obtain the ‘food parameter-specific DII score’. The ‘overall food parameter-specific inflammatory effect score’ was calculated based on the literature review conducted by Shivappa et al. [21] identifying the pro-inflammatory, anti-inflammatory, and null effects of each food parameter. Then, publication types were weighed according to study design for the pro-inflammatory and anti-inflammatory articles. For example, experimental studies in humans were assigned a higher weight than cross-sectional studies in humans. Third, the weighed publications were divided by the total number of articles. Next, the anti-inflammatory fraction was subtracted from the pro-inflammatory fraction. If the resulting number was higher or equal to 236, the food parameter was allocated the full value of the score. If the resulting number was lower than 236, it was first divided by 236 then multiplied by the food parameter-specific raw inflammatory effect score. Finally, all the ‘food parameter-specific DII scores’ were summed to create the ‘overall DII score’ for each woman in our study.

### 2.6. Statistical Analysis

Demographic characteristics were first examined by DII score tertile. Differences by tertile were identified using Kruskal–Wallis for continuous variables and chi-square test for categorical variables. Next, the association between DII score and SCM was evaluated by the Kruskal–Wallis test and SCM severity was tested by Mann–Whitney U-test. Generalized linear models of DII score in relation to SCM were fitted, adjusting for country and mode of delivery.

Multivariable regression was used to examine the association between nutrient intake in relation to SCM. Dietary intakes in our study population were compared to the Dietary Reference Values (DRV) of the European Food Safety Authority (EFSA) for lactating women [28]. The Average Requirement (AR) and Adequate Intakes (AI) were used to calculate the percentage of women with inadequate nutrient intakes. Logistic regression was used to compare the estimated odds ratio of being below the DRV among women without SCM and those with SCM, adjusting for country and mode of delivery as these 2 variables were associated with SCM [4] and DII score. The *p*-values were adjusted using the Benjamini and Hochberg correction and tested for significance at the α = 0.05 level. Analyses to estimate DII score were conducted using Python 3.6.8 (The Python Software Foundation, Fredericksburg, VA, USA) and inferential analyses were run with R version 3.3.2 (R Foundation for Statistical Computing, Vienna, Austria)

## 3. Results

The median DII score of the total population was 0.72, the 25th and 75th percentile values were −0.32 and 2.05, and the min and max were −3.52 and 4.07, respectively. Country and years of education were associated with DII (both *p* < 0.01). France (42%) and Portugal (43%) had the largest proportion of women in the highest DII tertile (Table 1). Overall, the proportion of women with more than 10 years of education was highest in DII tertile 1. 

**Figure 1:**
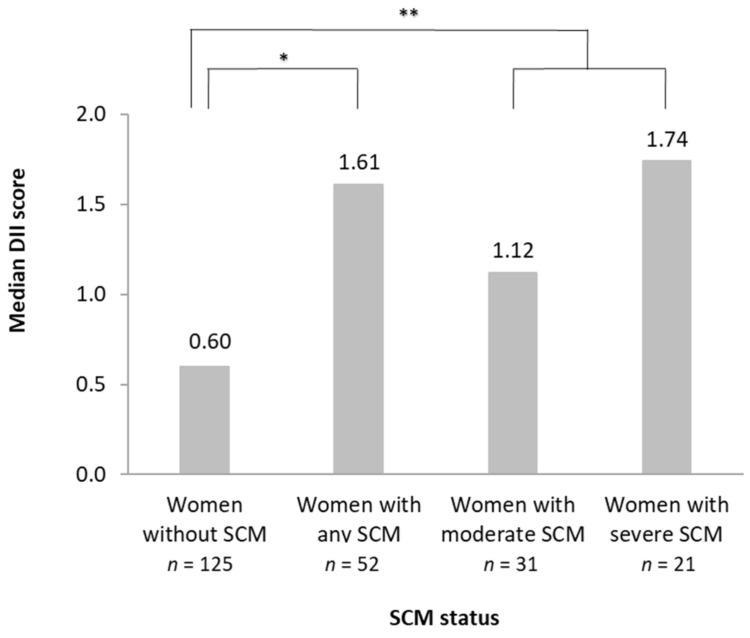
Median DII score by SCM status in lactating women from the ATLAS study (*n* = 177). * Comparison of women without SCM vs. any SCM performed with Mann–Whitney Test, *p* = 0.0055. ** Comparison of women without SCM vs. moderate or severe SCM performed with Kruskal–Wallis Test *p* = 0.0097. Women without SCM defined as Na:K ratio ≤0.6. Women with any SCM defined as Na:K ratio >0.6. Women with moderate SCM defined as Na:K ratio >0.6 and ≤1.0. Women with severe SCM defined as Na:K ratio >1.0. Standard deviations: no SCM = 1.71; any SCM = 1.82; moderate SCM = 1.86; severe SCM = 1.70. Abbreviations: DII, dietary inflammatory index; SCM, subclinical mastitis; NA:K, sodium potassium ratio.

A total of 29.4% (*n* = 52 out of 177) of women had at least one instance of SCM at any of the three visits (Figure 1). The overall prevalence of moderate SCM was 17.5% (*n* = 31) and severe SCM was 11.9% (*n* = 21). Of all SCM events, 40% were severe. 

Women with SCM had a significantly higher DII score than women without SCM (*p* < 0.01) (Figure 1). Women without SCM had a lower median DII (0.60) than women with moderate (1.12) or severe SCM (1.74) (*p* < 0.01) (Figure 1). The proportion of SCM increased from 25% in DII tertile 1 to 50% in tertile 3 (*p* = 0.03). A one-unit increase in DII was associated with about 41% increased odds of having SCM, adjusting for country and mode of delivery, *p* = 0.001.

Nutrient intakes in relation with SCM are shown in Table 2. In analyses adjusted for country and mode of delivery, women with SCM had significantly lower mean intakes of carbohydrates and fiber and nutrients that contribute to the normal function of the immune system (selenium, vitamin C, vitamin E, manganese), as well as magnesium, phosphorus, potassium, calcium, β-carotene, and B-vitamins (riboflavin, vitamin B6, folate, and vitamin B12) compared to women without SCM (*p* ≤ 0.05 for all listed nutrients). 

## 4. Discussion

In this study, for the very first time, we investigated the association between the inflammatory potential of the diet, using the DII, and SCM. We found that women without SCM had a lower median DII than women with moderate or severe SCM. The proportion of women with SCM increased from 25% in the first DII tertile to 50% in the third tertile. Women with SCM had lower mean intakes of several anti-inflammatory nutrients compared to women without SCM, although the odds of being below the DRV were not significant.

SCM was prevalent in almost one-quarter of lactating women at some timepoint during the first month after giving birth, which is comparable with observations made in other studies [29,30,31]. While the percentage of women below the DRV did not differ between groups, women with SCM in the current study had statistically significantly lower intakes of several minerals (potassium, magnesium, phosphorus, calcium, manganese, and selenium). Magnesium plays an important role in the immune system and animal studies demonstrate several benefits. In rodents, short-term magnesium deficient diets result in increased pro-inflammatory cytokines and decreased bifidobacteria content in the gut [32]. Mineral supplementation, particularly selenium supplementation, has been widely studied for maintaining dairy herd health and has consistently demonstrated a reduced risk of mastitis [33]. Selenium reduces lipid-damaging peroxides to protect cells from oxidative stress and has an antibacterial activity in milk [34]. Management of SCM prevention protocols has demonstrated that a multimineral injection (including selenium, copper, zinc, and manganese) did not reduce the incidence of SCM, but was associated with a lower prevalence of dairy cows with chronic clinical mastitis [35], while another study using the same injection protocol observed a reduction in the incidence of SCM (in all cows) and clinical mastitis (among multiparous cows) [36]. 

Women with SCM had significantly lower intakes of several vitamins (e.g., vitamins C and E, β-carotene, and several B vitamins) compared to women without SCM. These vitamins contribute to normal functioning of the immune system as well as reducing oxidative stress and the inflammatory response; however, the beneficial use of these vitamins for the prevention or treatment of SCM is not clear. In human immunodeficiency virus (HIV)-infected women, intake of both vitamin A and β-carotene supplements led to a higher risk of severe SCM [37]. Another study found that women with low body stores of vitamin A at baseline had higher Na:K ratios; however, 3 months of vitamin A supplementation did not affect Na:K ratio levels or reduce the increased mammary permeability [30]. Studies including vitamin E supplementation have shown more positive results. Dietary supplementation with sunflower oil rich in vitamin E lowered milk Na:K, as well as specific cytokines transforming growth factor β2 (TGF-β) and interleukin-8 (IL-8) at 3 months postpartum [38], suggesting a protective role of vitamin E for SCM. As an antioxidant, vitamin C supports the regeneration of vitamin E as an ion donor. Recent in vitro work has also demonstrated the role of vitamin C as a direct inhibiter of Gram positive bacterial growth, specifically Staphylococcus aureus [39], considered a main causative agent of mastitis [40]. In our cohort, the intake of several anti-inflammatory nutrients was low and the inflammatory potential of the diet was related to SCM. It could be hypothesized that the imbalance in the intake of anti-inflammatory and pro-inflammatory foods may be triggering oxidative stress that, in turn, may have a role to play in the etiology of SCM [41]. 

Our results support those findings that low intakes of vitamin E and C are associated with an increased risk for developing SCM. As with vitamin C, the vitamin B complex, of which we found four to be lower in the diet of women with SCM, have important roles in regulating the immune response, including B and T lymphocyte maturation and function [42]. In future studies, it would be beneficial to investigate the role of the vitamin B complex on the cytokine profile and the lymphocytes profiles of HM from women with SCM. Currently, the role of multi-vitamin supplementation to reduce the risk of SCM or alter inflammatory markers is limited and fails to demonstrate consistent effects. Hindle et al. reported no association between Na:K ratio or inflammatory markers in the HM of women supplemented with a multi-micronutrient supplement during pregnancy [43], while others using specific nutrient supplementation (vitamin A and vitamin E) during the lactation period reported a decrease in the HM Na:K ratio [38]. 

There are several limitations of this research. We were able to include only 27 of the 45 food parameters used to calculate DII due to limitations in the dietary assessment software. The missing food parameters could have improved our precision about the DII. Dietary data were collected only postpartum, therefore, we were unable to account for effects of the diet during late pregnancy. In addition, the study design did not allow us to establish causality between diet and SCM. While a poor diet could increase the risk for SCM, SCM may also change nutrient requirements. Furthermore, we did not collect data on how many of these women with elevated Na:K ratios eventually developed mastitis and if this impacted breastfeeding outcomes among these women. Women were also given the option to freely select which breast to use for sample collection. Given that SCM typically manifests unilaterally, it is possible that some cases were not detected. In addition, poor breastfeeding practices such as poor latch and not frequently emptying the breast may also contribute to SCM; however, breastfeeding practices were not evaluated in our study.

## 5. Conclusions

This is the first study to evaluate the relationship between DII and SCM. SCM was significantly associated with both DII and the dietary intake of micronutrients; further research should investigate the importance of these nutrients on SCM prevention and severity. The role of anti-inflammatory nutrients in reducing the risk for SCM should be evaluated in future studies with a larger sample size and include breastfeeding outcomes. This relationship needs to be tested in clinical trials in which a diet rich in antioxidants and anti-inflammatory nutrients could be compared to a standard diet to determine its utility in the prevention of SCM. The prevention of SCM is important for both maternal and infant health as SCM may impact the nutritional composition of HM [4]. In further research, it would be interesting to identify the histological changes taking place in breast tissue affected by SCM and to understand how these changes impact the transport of nutrients to HM.

## Figures and Tables

**Table 1:** Demographic characteristics by DII score tertile in lactating women from the ATLAS study (*n* = 177 ^a^).

	Tertiles of the DII			
Characteristic	T1 Median (Min, Max) −1.20 (−3.52, 0.06)	T2 Median (Min, Max) 0.70 (0.07, 1.67)	T3 Median (Min, Max) 2.22 (1.68, 4.07)	*p*-Value ^b^
Sample size, *n*	59	58	60	
Mean maternal age at delivery, years, mean (SD)	31.9 (3.4)	31.6 (4.4)	30.9 (3.9)	0.41
Country, *n* (%)				<0.01
Spain	2 (3)	7 (12)	0 (0)	
France	18 (31)	23 (40)	25 (42)	
Italy	7 (12)	2 (3)	5 (8)	
Norway	6 (10)	4 (7)	0 (0)	
Portugal	13 (22)	18 (31)	26 (43)	
Sweden	13 (22)	4 (7)	4 (7)	
Mean maternal pre-pregnancy BMI, kg/m^2^, mean (SD)	22.7 (2.2)	22.4 (2.4)	22.5 (2.9)	0.36
Number of years of education, *n* (%)				0.01
<10 years	17 (28)	35 (61)	26 (45)	
>10 years	42 (71)	22 (40)	32 (55)	
Mode of delivery, *n* (%)				0.30
Cesarean	6 (10)	10 (17)	12 (20)	
Vaginal	53 (90)	48 (83)	48 (80)	
Parity, *n* (%)				0.73
Primiparous	41 (69)	35 (60)	45 (75)	
Multiparous	18 (31)	23 (40)	15 (25)	

^a^ Sample size = 177 except for number of years of education *n* = 174. ^b^ Kruskal–Wallis and chi-square test for continuous and categorical variables, respectively. Abbreviations: DII, dietary inflammatory index; SD, standard deviation; BMI, body mass index; min, minimum; max, maximum.

**Table 2:** Daily energy and nutrient intakes and odds of being below the DRV by SCM status in lactating women from the ATLAS study (*n* = 177).

	Geometric Mean (95% CI) ^a^				Odds of Being below the DRV ^d^		
Nutrient Intake per Day	Women without SCM	Women with SCM	Difference ^b^	*p*-Value ^c^	Women without SCM	Women with SCM	*p*-Value ^e^
Energy, kcal	2125 (2010–2246)	1973 (1840–2114)	+152	0.07	2.7	4.2	0.85
Carbohydrates, g	113.5 (106.3–121.1)	110.7 (102–120.1)	+2.78	0.05	0.6	0.7	1
Proteins, g	41.2 (38.9–43.7)	41.3 (38.4–44.5)	−0.09	0.1	0	0.1	0.59
Fat, g	38.2 (35.7–40.9)	39.7 (36.5–43.2)	−1.46	0.52	0	0	1
Sugars, total, g	45.2 (41.4–49.5)	43.5 (38.9–48.7)	+1.73	0.09	0	0	1
Fiber, g	10.2 (9.3–11.1)	8.9 (7.9–9.9)	+1.32	0.01	3.3	5.5	0.27
SFA, g ^f^	14.6 (13.5–15.9)	15.5 (14–17.1)	+0.83	0.77	0.1	0.2	
MUFA, g ^f^	13.4 (12.4–14.4)	13.8 (12.5–15.1)	−0.37	0.46			
PUFA, g ^f^	4.9 (4.5–5.3)	5 (4.5–5.6)	−0.15	0.53			
Cholesterol, mg	139 (127.8–151.2)	147.8 (133.1–164.2)	-8.84	0.83			
18:2 *n*-6 (linoleic acid), g	3.2 (2.9–3.5)	3.3 (2.9–3.7)	−0.12	0.69	9.4	9.4	
18:3 *n*-3–3 (alpha linolenic acid), g	0.43 (0.39–0.48)	0.45 (0.4–0.52)	−0.02	0.77	6.4	3	
20:4 *n*-6 (arachidonic acid), g	0.05 (0.04–0.05)	0.06 (0.05–0.06)	−0.01	0.28			
20:5 *n*-3 (eicosapentaenoic acid), g	0.05 (0.04–0.08)	0.06 (0.04–0.1)	−0.01	0.77			
22:6 *n*-3 (docosahexaenoic acid), g	0.08 (0.06–0.11)	0.1 (0.07–0.14)	−0.01	0.74			
Sum *n*-6 fatty acids, g	1.65 (1.48–1.83)	1.73 (1.52–1.98)	−0.08	0.77			
Sum *n*-3 fatty acids, g	10 (9.3–10.8)	10.7 (9.7–11.8)	−0.66	0.84			
Vitamin A, µg RE	386.2 (337.1–442.4)	356 (300–422)	+30.43	0.12	0.16	0.08	0.48
β-carotene, µg	1161.5 (953.1–1415.4)	908 (709–1163)	+253.28	0.04			
Thiamin, mg	0.8 (0.7–1)	0.8 (0.6–0.9)	+0.08	0.08	0	0	1
Riboflavin, mg	0.9 (0.8–1.1)	0.9 (0.7–1)	+0.09	0.05	0.9	1.7	0.27
Niacin, mg	10.5 (9.3–11.7)	9.9 (8.6–11.5)	+0.53	0.14	0	0	1
Pantothenic acid, mg	2.8 (2.6–3.2)	2.6 (2.3–3)	+0.22	0.06	6.4	7.7	0.57
Vitamin B6, mg	1 (0.9–1.1)	0.9 (0.8–1)	+0.11	0.03	0.2	0.3	0.07
Folate, µg	175 (155.3–197.3)	154.8 (133.3–179.8)	+20.26	0.04	3	4.8	0.59
Vitamin B12, µg	2.8 (2.4–3.3)	2.4 (1.9–2.9)	+0.44	0.05	0.7	1.5	0.07
Vitamin C, mg	60.3 (50.5–71.9)	49.9 (40–62.3)	+10.35	0.05	2.3	4.8	0.59
Vitamin D, µg	2.4 (2–2.8)	2.4 (1.9–3)	−0.06	0.76	16.3	51	0.98
Vitamin E, mg	6.5 (5.6–7.4)	5.6 (4.7–6.7)	+0.85	0.05			
Calcium, mg	511.9 (460–569.5)	435.3 (380.9–497.5)	+76.56	0.01	0.3	1	0.16
Iron, total, mg	6 (5.2–6.9)	5.7 (4.7–6.7)	+0.37	0.19	0.2	0.3	0.4
Magnesium, mg	170 (155.4–186.1)	152.6 (136.3–170.8)	+17.43	0.01	0.9	1.6	0.16
Selenium, µg	73.7 (66.5–81.7)	66.4 (58.4–75.5)	+7.33	0.03	0.1	0.2	0.27
Phosphorus, mg	672.9 (632.7–715.8)	632.1 (585.1–682.8)	+40.87	0.01	0	0	1
Manganese, mg	1.6 (1.4–1.8)	1.3 (1.1–1.5)	+0.27	0.01	1.4	2.5	0.18
Copper, mg	0.8 (0.7–0.9)	0.7 (0.6–0.9)	+0.07	0.06	1.2	3.3	0.07
Zinc, mg	5.1 (4.6–5.6)	4.8 (4.2–5.4)	+0.31	0.07	1.5	2.5	0.74
Iodine, µg	77.3 (69–86.7)	74.3 (64.4–85.7)	+3	0.19	6.4	6.4	0.59
Sodium, mg	1270 (1160–1390)	1290 (1160–1450)	−20	0.5	0.2	0.4	0.37
Potassium, mg	1520 (1410–1640)	1390 (1260–1520)	+130	0.01	7.7	9.4	0.42

^a^ Geometric mean (95% CI) adjusted for energy (g or mg per 1000 kcal), country, and mode of delivery. ^b^ Difference in energy and nutrient intake between women without SCM and those with SCM. A positive (+) sign is used before the number when intake of a nutrient is numerically higher in the women without SCM than in women with SCM. A negative sign (−) is used when nutrient intake was numerically lower in women without SCM than women with SCM. ^c^ *p*-values adjusted using the Benjamini and Hochberg correction and tested for significance at the α = 0.05 level. ^d^ Estimated odds ratio of being below the DRV of women without SCM vs. those with SCM adjusting for country and mode of deliver using logistic regression analysis. Dietary intakes compared to the Dietary Reference Values (DRV) of the European Food Safety Authority (EFSA) for lactating women [28]. The Average Requirement (AR) and Adequate Intakes (AI) were used to calculate the percentage of women with inadequate nutrient intakes. Missing values are due to the fact that DRV are not available for all nutrients, for example MUFA, PUFA, and cholesterol. ^e^ *p*-values adjusted using the Benjamini and Hochberg correction and tested for significance at the α = 0.05 level. ^f^ Abbreviations: SFA = saturated fatty acids, MUFA = monounsaturated fatty acids; PUFA = polyunsaturated fatty acids. SCM, subclinical mastitis; CI, confidence interval; RE, retinol equivalents.

## Data Availability

Not applicable.
